# Clinical and swallowing characteristics of tracheostomized patients with post-intubation acquired tracheal or laryngotracheal stenosis^✡^

**DOI:** 10.1016/j.clinsp.2024.100552

**Published:** 2024-12-07

**Authors:** Carina Escudero, Fernanda Chiarion Sassi, Ana Paula Ritto, Paulo Francisco Guerreiro Cardoso, Claudia Regina Furquim de Andrade

**Affiliations:** a Departamento de Fisioterapia, Fonoaudiologia e Terapia Ocupacional da Faculdade de Medicina da Universidade de São Paulo, São Paulo, SP, Brazil; b Departamento Cardiopneumologia, Disciplina de Cirurgia Torácica da Faculdade de Medicina da Universidade de São Paulo, São Paulo, SP, Brazil

**Keywords:** Tracheostomy, Laryngotracheal stenosis, Subglottic stenosis, Dysphagia, Risk factors

## Abstract

**Objective:**

To investigate the clinical and swallowing profiles of patients with post-intubation tracheal or laryngotracheal stenosis requiring tracheostomy prior to surgical or corrective airway interventions.

**Methods:**

A retrospective cohort clinical study. Patients who met the inclusion criteria underwent a swallowing evaluation, as well as imaging studies of the neck and chest. At 6 and 12-months after the initial assessment, all patients participated in follow-up speech pathology consultations to evaluate the current state of swallowing and the progress of airway stenosis treatment.

**Results:**

Twenty-five patients with a median tracheostomy duration of 30.52 months were assessed. The study found a female predominance (68%) and a high prevalence of subglottic stenosis (44%). Most patients required intubation due to severe acute respiratory failure and airway protection due to a decreased level of consciousness. Imaging revealed additional airway abnormalities, including laryngotracheobronchitis and laryngeal edema. Swallowing assessments showed that 20% had significant dysphagia. After one year, only two patients were successfully decannulated, while others remained tracheostomy dependent.

**Conclusion:**

The study underscores the need for personalized, multidisciplinary care for these patients. It finds that while treatments like reconstructive surgeries and the Montgomery T-tube can be effective, complications such as clinical instability and dysphagia can worsen outcomes and extend the need for tracheostomy.

## Highlights

• Despite the efficacy of reconstructive surgeries and Montgomery T-tube, clinical and neurological instability significantly impact outcomes and prolong tracheostomy use.
• Treatment for post-intubation tracheal or laryngotracheal stenosis requires a multidisciplinary and personalized approach.
• Dysphagia is a significant factor linked to poorer clinical outcomes and extended tracheostomy use.

## Introduction

Laryngeal and tracheal stenosis are rare conditions characterized by the narrowing of the upper airway, leading to symptoms such as dyspnea, stridor, and dysphonia.^1^ In adults, the etiology is diverse and often linked to prolonged Endotracheal Intubation (ETI), tracheostomy, post-treatment complications of head and neck tumors, as well as autoimmune, inflammatory, or idiopathic conditions.^2^ According to the literature, around 0.20% of patients requiring ETI develop benign upper airway stenosis.^3^ This condition is commonly linked to prolonged intubation duration,^3^ improper positioning of the orotracheal tube, and excessive cuff inflation.^1^

Patients with airway stenosis may require tracheostomy, stent implantation, or various surgical approaches for correction.^4^ In this process, long-term dependence on tracheostomy poses significant emotional, practical, and social challenges for patients with airway stenosis and their families.^5^ Additionally, difficulties in managing tracheostomy, prolonged use of the device, and underlying conditions can lead to dysphagia,^6^ which compromises the success of clinical treatment.^7^ Dysphagia increases the risk of active infections,^7^ potentially altering the airway microbiome and inducing hyperresponsiveness, thereby exacerbating stenosis.^3^ Moreover, dysphagia can result in malnutrition, dehydration, and an increased risk of pneumonia,^8^ worsening the patients' clinical condition and consequently hindering the feasibility of corrective surgeries.

Despite its importance, the literature on dysphagia in tracheostomized patients with airway stenosis is limited and tends to focus on the duration of symptoms after interventions, even though preoperative nutritional status is a crucial indicator of postoperative outcomes and overall recovery.^9^ In the context of care for tracheostomized patients, the presence of dysphagia is an indicator of decannulation failure, whereas early swallowing assessment and improvement in functional swallowing level are associated with successful decannulation processes.^10^

This study focused on the clinical and swallowing profile of patients with benign post-intubation tracheal or laryngotracheal stenosis wearing a tracheostomy prior to surgical or corrective airway interventions. The study also identified the contributing factors to tracheostomy dependency in such patients to help plan further therapeutic strategies.

## Material and methods

This is a retrospective observational clinical study involving patients with benign tracheal or laryngotracheal stenosis post-intubation who underwent tracheostomy and were treated from November 2020 to June 2023 at the Division of Oral Myology, Hospital das Clínicas, School of Medicine, University of São Paulo, Brazil. The study received approval from the local Institutional Review Board (CAPPesq HCFMUSP - Process n° 4,859,177).

### Participants

Participants in this study were referred from specialized outpatient clinics (e.g., Airway Surgery, Otorhinolaryngology, Head and Neck Surgery) or following discharge from the same quaternary-level hospital.

Inclusion criteria: patient requiring tracheostomy; 16-years or older; diagnosis of post-intubation benign laryngeal and/or tracheal stenosis; no previous history of airway reconstruction surgery.

Exclusion criteria: in use of mechanical ventilation or supplemental oxygen catheter; presence of a tracheoesophageal fistula; Glasgow Coma Scale score < 9; diagnosis of progressive neurological and/or neuromuscular diseases; presence of tumor, infection, or foreign body in the upper airway.

According to the protocols established at our institution, the medical team evaluates patients wearing a tracheostomy, followed by imaging studies such as neck and chest computed tomography scans and flexible bronchoscopy. The objective is to evaluate the stenosis's degree, extent, and precise location to determine the most suitable treatment approach. Treatment options encompass surgical resection with definitive tracheal or laryngotracheal reconstruction, insertion of a silicone T-tube, or a silicone endotracheal stent.^11^ Contraindications for definitive surgical treatment of tracheal stenosis include stenosis over 50 mm in length, severe cognitive impairment, severe cardiovascular disease; the need for continuous mechanical ventilation, the need for a surgical procedure that requires ETI and general anesthesia.^11^

### Procedure

Patients who met the inclusion criteria underwent a speech pathology evaluation, consisting of clinical and swallowing parameters. Clinical data included variables such as age, gender, comorbidities, Glasgow Score^12^ and location of the stenosis (laryngeal, subglottic, laryngotracheal, suprastomal trachea, or infrastomal trachea). The need for ETI was categorized as airway protection when there was a decrease in the consciousness level, acute respiratory failure, or inadequate ventilation/oxygenation. Other underlying diagnoses associated with ETI, intubation duration, and tracheostomy duration were also considered.

The diagnosis of airway stenosis was confirmed by laryngotracheobronchoscopy and/or neck and chest computed tomography. Reports included data on the presence or absence of any other structural airway abnormalities, such as laryngeal edema, laryngotracheobronchitis, airway collapse (either structural tracheal collapse such as in tracheal malacia, or excessive dynamic airway collapse of the posterior tracheal wall), vocal cord paresis or paralysis, obstructive granuloma, and/or associated sub-stenosis.

The chest CT scan looked for other pulmonary conditions such as emphysema, ground-glass opacities, reticular opacities, fibrosis, micronodules, consolidation, bronchial thickening, and atelectasis.

Clinical severity was determined using the Charlson Comorbidity Index,^13^ which consists of twenty clinically significant conditions empirically weighted to predict patient mortality risk and stratified into the following scores: 3 = Low risk; 4 to 5 = Moderate risk; 6 to 7 = High risk; > 8 = Very high risk.

### Swallowing function assessment

The evaluation of swallowing function began by assessing the possibility of cuff deflation. Subsequently, a digital occlusion tolerance test during phonation was conducted for patients who maintained clinical stability with the cuff deflated. This test was performed on patients who came for the speech pathology evaluation with either a deflated or uncuffed tracheostomy tube. Clinical instability was defined by the onset or worsening of dyspnea, persistent or uncontrollable cough, and/or a decrease in the oxygen saturation over 5% compared to baseline baseline. In such cases, the cuff was inflated again, and the assessment concluded. The evaluation continued per protocol in patients with an unobstructed tracheostomy tube and sustained clinical stability.

#### Dysphagia risk evaluation protocol (DREP)

The DREP[14] is a Brazilian protocol validated and designed to assess the risk of dysphagia by administering controlled volumes (3 mL, 5 mL, and 10 mL) and free volumes (50 mL) of water and homogeneous pasty food, with three repetitions each. Results are recorded as “pass” or “fail”, and administration is stopped if the patient shows clinical signs suggesting laryngotracheal penetration or aspiration. The DREP screening version demonstrated excellent validity with sensitivity at 92.9%, specificity at 75.0%, negative predictive values at 95.5%, and an accuracy of 80.9%.^14^

In a study by Medeiros et al.^15^ involving patients with prolonged ETI, indicators such as multiple swallows, altered cervical auscultation, post-swallowing vocal quality changes, coughing, and choking were identified as risks for bronchial aspiration. This study considered only coughing, choking, multiple swallows, and altered cervical auscultation since some participants could not phonate due to upper airway obstruction.

#### Protocol for introduction and transition of feeding (PITF)

Based on the American Dietetic Association model, the PITF^16^ is designed to assess swallowing with foods and liquids of varying consistencies and larger volumes. Its methodology integrates signs and symptoms commonly encountered in clinical speech pathology practice, helping to determine safe oral feeding decisions.

#### Blue dye test

The blue dye test^17^ is a widely used technique for detecting salivary aspiration in tracheostomized patients. It involves administering four drops of blue food dye into the patient's oral cavity using a syringe or spoon. Subsequently, with the cuff deflated, the patient is instructed to swallow saliva, and the presence or absence of blue dye in the tracheostomy site is observed. The detection of blue dye indicates a positive result for salivary aspiration. The test duration is four hours, including evaluation of delayed outcomes.

Additionally, the modified blue dye test assesses the potential for liquid and food aspiration. In this procedure, blue food dye is mixed with water or homogeneous pasty foods to evaluate swallowing function. The presence of blue dye inside or around the tracheostomy suggests the presence of aspiration events.

#### Functional level of swallowing

To assess the functional level of swallowing, the authors utilized the Functional Oral Intake Scale from the American Speech-Language-Hearing Association National Outcome Measurement System (ASHA NOMS ‒ 2003).^18^ This multidimensional scale assigns a number from 1 to 7 based on the supervision needed for feeding and diet (Table 1). The ASHA NOMS scale categorizes individuals based on their ability to safely swallow and manage diets, aiding in treatment planning and monitoring of dysphagia interventions.

**Table 1:** National Measurement System Results – American Speech-Language-Hearing Association – National Outcomes Measurement System – ASHA NOMS.

ASHA-NOMS scale	
**LEVEL 1:**	Individual is not able to swallow anything safely by mouth. All nutrition and hydration is received through non-oral means (e.g., nasogastric tube, PEG).
**LEVEL 2:**	Individual is not able to swallow safely by mouth for nutrition and hydration, but may take some consistency with consistent maximal cues in therapy only. Alternative method of feeding is required.
**LEVEL 3:**	Alternative method of feeding required as individual takes less than 50% of nutrition and hydration by mouth, and/or swallowing is safe with consistent use of moderate cues to use compensatory strategies and/or requires maximum diet restriction.
**LEVEL 4:**	Swallowing is safe, but usually requires moderate cues to use compensatory strategies, and/or the individual has moderate diet restrictions and/or still requires tube feeding and/or oral supplements.
**LEVEL 5:**	Swallowing is safe with minimal diet restriction and/or occasionally requires minimal cueing to use compensatory strategies. The individual may occasionally self-cue. All nutrition and hydration needs are met by mouth at mealtime.
**LEVEL 6:**	Swallowing is safe, and the individual eats and drinks independently and may rarely require minimal cueing. The individual usually self-cues when difficulty occurs. May need to avoid specific food items (e.g., popcorn and nuts), or require additional time (due to dysphagia).
**LEVEL 7:**	The individual's ability to eat independently is not limited by swallow function. Swallowing would be safe and efficient for all consistencies. Compensatory strategies are effectively used when needed.

After completing the speech pathology assessment, speech pathology management was defined as a referral for swallowing rehabilitation or assisted speech pathology discharge. Patients referred for swallowing rehabilitation underwent a program based on orofacial motor and swallowing training. It included indirect therapy focusing on muscle coordination and strength, management of oral secretions, cuff manipulation, and training of the respiratory-swallow coordination.^19^^,^^20^ Direct therapy involves food-based interventions.^19^

At 6 and 12 months post-assessment all patients underwent follow-up speech pathology consultations for assessment of the current feeding status and swallowing-related complaints. Based on these consultations and medical records, clinical outcomes were classified as follows: decannulation post-surgery, silicone T-tube use, ongoing medical treatment with tracheostomy, continued tracheostomy use without surgical candidacy, and mortality. The swallowing functional levels were also reassessed according to the ASHA NOMS scale.

## Results

The Division of Oral Myology evaluated forty-three patients with airway stenosis. Ten patients with airway stenosis associated with head and neck tumors, 2 with idiopathic tracheal stenosis, 1 with laryngeal amyloidosis, and 5 who had undergone a previous surgical intervention for airway stenosis were excluded. The study population consisted of 25 patients with tracheostomy diagnosed with post-intubation tracheal or laryngotracheal stenosis with a mean duration of 30.52 months (median 7 months, range 1–204 months).

Table 2 shows the clinical and demographic data of the study population. It is observed a female predominance (68%) and a predominance of subglottic laryngeal stenosis diagnoses (44%). The main causes associated with ETI were severe acute respiratory failure (48%) and airway protection due to a decreased level of consciousness (40%). These findings correlated with COVID-19 (28%) and neurological diseases (32%) including Traumatic Brain Injury (TBI) and stroke. The median Charlson Comorbidity Index for the entire cohort was 1.00 (indicating a low risk of death). The most common comorbidities were systemic arterial hypertension (68%) and diabetes mellitus (40%).

**Table 2:** Clinical and demographic characterization (n = 25).

Age (years), Median (P25; P75)		58 (25; 75)
**Gender, n (%)**	Male	8 (32%)
	Female	17 (68%)
**Glasgow, Median (P25; P75)**		15 (9; 15)
**Charlson Comorbidity Index, Median (P25; P75)**		1.0 (0.0; 8.0)
**Location of stenosis, n (%)**	Laryngotracheal	5 (20%)
	Subglottic laryngeal	11 (44%)
	Suprastomal tracheal stenosis	8 (32%)
	Infrastomal tracheal stenosis	1 (4%)
**Cause of ETT, n (%)**	Airway protection due to decreased level of consciousness	10 (40%)
	Acute Respiratory Failure	12 (48%)
	Inadequate oxygenation or ventilation	3 (12%)
**Medical diagnosis related to the cause of ETT, n (%)**	COVID-19	7 (28%)
	Stroke	3 (12%)
	Traumatic Brain Injury/Polytrauma	5 (20%)
	Acute Myocardial Infarction	2 (8%)
	General Surgery	3 (12%)
	Other clinical conditions	5 (20%)
**Duration of ETI (days), Median (P25; P75)**		19 (4; 35)

n, number of patients; ETI, Endotracheal Tube; P25, 25^th^ percentile; P75, 75^th^ percentile.

Table 3 presents the results of airway imaging assessments, highlighting anatomical changes beyond the stenosis. In the larynx and trachea, 20% of patients had laryngotracheobronchitis and 16% exhibited laryngeal edema. Chest CT scans revealed the clinical complexity of the patients, evidenced by various associated pulmonary abnormalities, including a high incidence of pulmonary atelectasis, micronodules, and ground-glass opacities.

**Table 3:** Results of airway imaging assessments.

Imaging assessment	Anatomical changes	n (%)
Imaging assessment (airway endoscopy and neck CT scan)	No other alterations besides stenosis	12 (48%)
	Laryngeal edema	4 (16%)
	Laryngotracheobronchitis	5 (20%)
	Anterior tracheal collapse	1 (4%)
	Paresis/paralysis of vocal folds	2 (8%)
	Obstructive granuloma	1 (4%)
Chest CT scan	Normal	3 (12%)
	Emphysema	4 (16%)
	Ground-glass opacities	8 (32%)
	Reticular opacities	3 (12%)
	Fibrosis	1 (4%)
	Micronodules	9 (36%)
	Lung opacity and bronchial wall thickening	8 (32%)
	Atelectasis	10 (40%)

n, Number of participants; P25, 25^th^ percentile; P75, 75^th^ percentile.

Table 4 shows the outcomes of patients at 6- and 12-months following speech-language pathology evaluation. After 6 months, one patient died due to abdominal sepsis, and 76% remained dependent on tracheostomy. Out of the 24 patients followed up at one year, 10 (41.67%) met the criteria for reconstructive surgeries, with 2 (8.33%) successfully decannulated post-surgery. The medical team opted for treatment using the Montgomery T-tube for 10 patients (41.67%), resulting in successful decannulation for one patient following treatment.

**Table 4:** Outcome at 6- and 12-months.

Outcome	6-months follow-up (n = 25)	12-months follow-up (n = 24)
Tracheostomy, airway endoscopic procedures	13 (52%)	8 (33.3%)
Wearing a silicone T-tube	5 (20%)	9 (37.5%)
Underwent reconstructive surgery and decannulation	0 (0%)	2 (8.3%)
Remained with a tracheostomy, not eligible for surgery	6 (24%)	5 (20.9%)
Death	1 (4%)	0 (0%)

n, Number of participants; P25, 25^th^ percentile; P75, 75^th^ percentile.

Patients managed over one year, 25% were ineligible for surgery and remained with a tracheostomy. The contraindications for surgery included neurological (66.67%), or clinical instability with multiple comorbidities (33.33%).

Regarding swallowing assessments, the authors found 3 patients with abnormal saliva, in terms of organization and frequency. These patients underwent the blue dye test, and 2 patients tested positive for saliva aspiration. Among the 23 patients who underwent the dysphagia screening test (DREP), 1 was identified as at risk for dysphagia in the thin liquid test (cough), 1 patient tested positive for aspiration of thin liquid in the modified blue dye test, despite the absence of cough, choking, throat clearing, or changes in cervical auscultation, suggesting silent aspiration. Of the 22 patients considered as suitable for oral-only feeding, 8 (36.3%) exhibited abnormalities in mastication and swallowing of semi-solid and solid foods, whereas the other 14 patients showed no significant abnormalities.

Table 5 shows data related to swallowing function. During the initial assessment, 20 patients (80%) were identified with functional or normal swallowing (ASHA NOMS 6 and 7), whereas 5 patients (20%) had several degrees of dysphagia (ASHA NOMS 1 to 5), where 3 patients (12%) were on an alternative feeding. Of the 5 dysphagic patients, at one-year follow-up, one had passed away, 3 who were ASHA NOMS 1 continued to experience the same swallowing difficulties, and 1 patient progressed to functional swallowing following speech therapy rehabilitation.

**Table 5:** Distribution of patients among the different functional levels of swallowing (ASHA NOMS).

Level	Baseline assessment (n = 25)	6-months follow-up (n = 24)	12-months follow-up (n = 24)
1	1 (4%)	1 (4.1%)	1 (4.1%)
2	1 (4%)	1 (4.1%)	1 (4.1%)
3	1 (4%)	1 (4.21)	1 (4.1%)
4	0 (0%)	0 (0%)	0 (0%)
5	2 (8%)	1 (4.21)	1 (4.1%)
6	6 (24%)	6 (25%)	5 (20.8%)
7	14 (56%)	14 (58.3%)	15 (62.5%)

n, Number of participants; P25, 25^th^ percentile; P75, 75^th^ percentile; ASHA NOMS, Functional level of swallowing according to the American Speech-Language-Hearing Association National Outcome Measurement System.

In the analysis of patients with dysphagia who were classified between ASHA NOMS levels 1 and 5, the median age was 65 years, and the median duration of tracheostomy use was 8 months. Predominant underlying conditions included neurological diseases (3 patients) that required ETI due to lowered level of consciousness, and 2 patients for inadequate oxygenation following surgery. The median Charlson Comorbidity Index was 3, and the Glasgow Coma Scale score was 11 points. Stenosis classification revealed three patients with supra-stomal tracheal stenosis, one with subglottic stenosis, and one with infra-stomal stenosis. Among the 4 dysphagic patients managed beyond 12 months, all remained with a tracheostomy. Three were not suitable for definitive surgery because of their severely compromised clinical status.

## Discussion

The present study comprehensively analyzes patients with tracheostomy diagnosed with post-intubation tracheal or laryngotracheal stenosis. The results emphasize the need for a personalized and multidisciplinary approach to treating and rehabilitating these patients. Furthermore, the presence of dysphagia may be associated with poor clinical outcome, particularly with prolonged tracheostomy use.

In the present cohort, the median time of tracheostomy use before the onset of specialized treatment was 7 months, reflecting the chronic and complex nature of these cases. This situation is possibly influenced by the clinical profile of the institution, which is an academic quaternary public hospital in which most patients require clinical stabilization following previous care at lower-complexity centers.

Corroborating this reality, the study by Singh et al.^2^ described the characteristics and outcomes of patients with laryngotracheal stenosis, demonstrating a wide spectrum of clinical manifestations in this population. According to the authors, this diversity complicates the standardization of treatment and results in variability in therapeutic outcomes, influenced by the complexity of the disease pathogenesis, missed medical appointments, complex surgeries, and personal preferences regarding surgical intervention.

Another finding of this study was the predominance of females (68%) in the studied population, in accordance with Johnson et al.,^3^ who suggested a higher incidence of airway stenoses in women. This may be associated with a stronger immune response for tissue repair, in addition to a higher likelihood of intubation with an inadequately sized endotracheal tube.^3^

Regarding the clinical status of the patients, the median Charlson Index score of 1.0 indicated a low risk of mortality. However, long-term follow-up highlights that clinical instability can present significant challenges in managing patients with airway stenosis due to the risks associated with surgical interventions and potential postoperative complications. There is a need for healthcare teams to engage in early discussions with patients and their families to ensure appropriate future planning. The high prevalence of hypertension (68%) and diabetes mellitus (40%) among the patients is consistent with existing literature, which links these conditions to increased systemic inflammation and impaired wound healing.^21^ These factors elevate the risk of postoperative complications and may eventually contraindicate surgical intervention.^21^

The present study revealed that approximately half of the patients exhibited additional upper airway abnormalities beyond stenosis. Specifically, laryngotracheobronchitis (20%) and laryngeal edema (16%) were prevalent among these abnormalities. These findings are consistent with the systematic review and meta-analysis by Brodsky et al.,^22^ which examined laryngeal injuries in patients undergoing ETI. The meta-analysis indicated that most patients with laryngeal injuries had soft tissue changes, such as glottic edema and tracheal irritation. Conversely, a smaller subset of patients presented with more severe injuries, including vocal fold paralysis, ulcerations, and fibrin deposition, leading to functional complications, particularly dysphonia.

Regarding the pulmonary health status of the studied patients, imaging studies revealed a high incidence of abnormalities such as atelectasis and ground-glass opacities. These changes often occur due to secondary infections and inflammations, reduced cough efficiency, secretion retention, and decreased lung capacity.^23^ These findings reinforce the clinical complexity of patients with upper airway stenosis, highlighting the necessity for comprehensive and continuous evaluation to tailor treatment plans effectively in a personalized and multidisciplinary manner.

Considering the results of this clinical follow-up, the authors found that only two patients were successfully decannulated within a year. This observation is consistent with the literature, which reports prolonged tracheostomy use and high risks associated with airway reconstruction surgeries due to the complexity and recurrence risk of stenosis, leading to a high propensity for tracheostomy dependence in this population.^2^ Adopting the Montgomery T-tube in 41.67% of patients highlights its role as a valuable intervention procedure, frequently pointed out in the literature as an effective temporary management strategy while evaluating other definitive interventions, mainly due to its facilitation of phonation.^4^

In the present study, concerning the functional status of swallowing, speech pathology assessments revealed that 80% of patients had functional or normal swallowing, while 20% presented with dysphagia. Additionally, patients with moderate to severe dysphagia had a median age of 65 years and a more severe clinical condition, possibly associated with neurological impairments, as reflected by a median Charlson Index of 3 and Glasgow Coma Scale score of 11 points. These findings suggest that dysphagia may be more closely linked to patients' clinical conditions than to the use of tracheostomy or the presence of airway stenosis, consistent with the systematic review by Skoretz et al.^6^ Additionally, the association between dysphagia and aging is documented in other studies, such as Leira et al.,^24^ which found a relationship between dysphagia in elderly individuals and cognitive impairment, functional dependency, and loss of muscle strength.

Among the patients with dysphagia in this study, one passed away, and all others continued to have a tracheostomy throughout one year. After speech therapy rehabilitation, only one patient progressed to functional swallowing, while three continued to have the same dysphagia condition. These results highlight that complete recovery of swallowing functionality may be limited and dependent on multiple clinical factors, although intensive speech therapy may be effective in some cases. The scientific literature on the management of tracheostomized patients with chronic dysphagia is limited, revealing a significant gap in research and clinical practice. Studies suggest that chronic dysphagia and prolonged use of alternative feeding routes may be associated with an increased risk of comorbidities and mortality.^25^ The present study also identified that the presence of dysphagia is a potential marker for worse clinical outcomes and prolonged use of tracheostomy. It can be explained by the aspiration of saliva, which promotes the colonization and inhalation of pathogenic bacteria in the oropharynx and respiratory tract, resulting in endogenous infections^26^ and poorer mucosal healing, thereby aggravating stenosis.^3^ In this context, preventing aspiration pneumonia requires a multifaceted approach, including swallowing rehabilitation therapy, pharmacological treatment, oral hygiene, and proper positioning, especially in elderly patients.^26^

This study presented a few limitations. All participants were recruited from a single institution, resulting in a small sample size that can affect the generalizability of the study findings. However, the authors mitigated selection bias by employing a standardized evaluation protocol and ensuring sample homogeneity by including only patients with benign post-intubation tracheal stenosis. Despite these limitations, this study provided more profound insights into the complexities associated with airway stenosis. It underscored the importance of personalized approaches for the treatment and rehabilitation of these patients.

## Conclusion

The current study's findings emphasize the essential requirement for thorough, multidisciplinary management of patients with airway stenosis. While treatment strategies such as reconstructive surgeries and the utilization of the Montgomery T-tube have shown efficacy in some instances, challenges such as clinical and neurological instability, along with the presence of dysphagia, significantly impact therapeutic outcomes and necessitate prolonged tracheostomy use. The presence of dysphagia emerged as a potential indicator for worse clinical outcomes.

## Authors’ contributions

Carina Escudero: Responsible for data collection and analysis; interpretation of the results; writing major portion of the paper.

Fernanda Chiarion Sassi: Responsible for supervising the research; interpretation of the results; writing major portion of the paper.

Ana Paula Ritto: Contributed to data analysis and interpretation; organized the statistical analyses; contributed to manuscript preparation.

Paulo Francisco Guerreiro Cardoso: Contributed to data analysis and interpretation; responsible for revising the final version of the manuscript.

Claudia Regina Furquim de Andrade: Responsible for the research and experimental design; contributed to data analysis and manuscript preparation.

## Funding

None.

## Declaration of competing interest

The authors declare no conflicts of interest.
